# Homo-FRET Based Biosensors and Their Application to Multiplexed Imaging of Signalling Events in Live Cells

**DOI:** 10.3390/ijms160714695

**Published:** 2015-06-30

**Authors:** Sean C. Warren, Anca Margineanu, Matilda Katan, Chris Dunsby, Paul M. W. French

**Affiliations:** 1 Photonics Group, Department of Physics, Imperial College London, London SW7 2AZ, UK; E-Mails: a.margineanu@imperial.ac.uk (A.M.); christopher.dunsby@imperial.ac.uk (C.D.); paul.french@imperial.ac.uk (P.M.W.F.); 2 Structural and Molecular Biology, University College London, London WC1E 6BT, UK; E-Mail: m.katan@ucl.ac.uk

**Keywords:** time resolved fluorescence anisotropy imaging (TR-FAIM), FRET, multiplexed imaging, homo-FRET

## Abstract

Multiplexed imaging of Förster Resonance Energy Transfer (FRET)-based biosensors potentially presents a powerful approach to monitoring the spatio-temporal correlation of signalling pathways within a single live cell. Here, we discuss the potential of homo-FRET based biosensors to facilitate multiplexed imaging. We demonstrate that the homo-FRET between pleckstrin homology domains of Akt (Akt-PH) labelled with mCherry may be used to monitor 3′-phosphoinositide accumulation in live cells and show how global analysis of time resolved fluorescence anisotropy measurements can be used to quantify this accumulation. We further present multiplexed imaging readouts of calcium concentration, using fluorescence lifetime measurements of TN-L15-a CFP/YFP based hetero-FRET calcium biosensor-with 3′-phosphoinositide accumulation.

## 1. Introduction

Multiplexing of Förster resonant energy transfer (FRET) readouts utilising genetically expressed biosensors incorporating fluorescent proteins provides opportunities to correlate multiple signalling pathways in live cells in space and time. However, the lack of suitable practical donor–acceptor fluorescent protein combinations in the red spectral region has limited the application of multiplexed FRET biosensors in live cells to date. Homo-FRET is the phenomenon of Förster resonant energy transfer between identical fluorophores. It has the potential to be more spectrally efficient than hetero-FRET and so is interesting for multiplexed readouts of protein interactions. Here we present a red homo-FRET readout realised using time resolved fluorescence anisotropy imaging (TR-FAIM) of mCherry and use this with TN-L15-an enhanced cyan fluorescent protein (ECFP)-yellow fluorescent protein (YFP) biosensor for calcium-to demonstrate the multiplexed readout of two FRET biosensors in a live cell. The development of a polarisation resolved time-correlated single photon counting (TCSPC) microscope system for multiplexed imaging of hetero- and homo-FRET sensors is described below together with the use of global analysis to obtain quantitative information about clustering parameters on a pixel-by-pixel basis from low photon count data. These techniques have been applied to read out accumulation of PtdIns(3,4,5)*P*_3_ and PtdIns(3,4)*P*_2_ (3′-phosphoinositides) at the membrane using an Akt-PH based homo-FRET sensor and measurements of 3′-phosphoinositide accumulation multiplexed with a calcium biosensor.

### 1.1. Approaches to Multiplexed Förster Resonant Energy Transfer (FRET) Measurements

A number of approaches to multiplexed imaging of FRET biosensors have been previously demonstrated, exploiting the spectral diversity of fluorescent proteins, for example, employing blue-yellow and orange-red pairs whose emission can be spectrally separated.

Spectral ratiometric imaging of two spectrally interleaved FRET pairs was demonstrated by Niino *et al.* using biosensors tagged with Sapphire-RFP and ECFP-YFP to monitor intracellular cyclic guanosine monophosphate (cGMP) and cyclic adenosine monophosphate (cAMP) respectively [1]. Emission was measured in four spectral channels and a linear unmixing procedure was used to separate emission from each of the fluorophores to overcome the high degree of crosstalk across the spectral channels. Similarly, Piljic *et al.* applied spectral ratiometric imaging to FRET biosensors tagged with a mOrange-mCherry pair and a ECFP/YFP pair to monitor cytosolic calcium, membrane-bound protein kinase C (PKC) activity and annexin A4 [2]. In this work both calcium and PKC probes were tagged using ECFP/YFP and the distinct spatial localisation of the two probes within the cell was exploited to discriminate their responses. This quad spectral channel approach suffers from high levels of noise introduced by the data processing required to remove crosstalk between the fluorophores and requires a number of auxiliary experiments to determine the emission spectra of the individual fluorophores.

In previous work we demonstrated a different approach using a hybrid spectral ratiometric/FLIM multiplexing methodology [3]. Here, fluorescence lifetime imaging (FLIM) was used to report the activity of a Raichu-Ras probe, with TagRFP as the donor with mPlum acting as an almost dark acceptor, while spectral ratiometric imaging was employed in parallel to read out an ECFP-Venus tagged chameleon Ca^2+^ sensor. Compared to quad channel ratiometric imaging, this approach offers a better separation of the two biosensors since the use of FLIM means that a low quantum efficiency fluorophore may be used as the acceptor (since the acceptor fluorescence is not measured). In particular, it is possible to pair low efficiency deep red fluorophores such as mPlum with RFP donors, thereby realising a significantly greater spectral separation from ECFP-YFP. This particular implementation by Grant *et al.*, however, was compromised by the rapid photobleaching of TagRFP that limits its use for time course experiments. Subsequently TagRFP-T, an improved variant of TagRFP reported to significantly improved photostability [4], was incorporated in a TagRFP-T-mPlum FRET biosensor of calcium concentration based on the ECFP-YFP probe TN-L15 [5]. The lifetime of TagRFP-T was, however, found to be highly sensitive to photobleaching under pulsed excitation in a Nipkow disc microscope, decreasing from 2.2 to 1.78 ns over ten minutes [5]. A later comprehensive study of the photo-physics of red fluorescent proteins [6] indicated that dark state conversion and irreversible photobleaching mechanistic pathways vary across different RFP species and in some RFP can lead to differential photobleaching decay rates depending on the intensity and duration of excitation. These authors found only two RFP with photobleaching properties under realistic illumination conditions that approach those of EGFP: mCherry and TagRFP R67K S158T-a novel mutant of TagRFP [6]. mCherry has been used extensively in fluorescence studies and its properties are reasonably well understood [7,8] while TagRFP R67K S158T, however, has not yet been used by other groups. Since TagRFP R67K S158T is not yet fully characterised and may present other drawbacks in practice, it seems desirable to utilise mCherry in longer wavelength FRET biosensors to enable multiplexing with the wide range of ECFP-YFP based probes. Unfortunately, the large Stokes shift of mCherry precludes its use as a hetero-FRET donor with an acceptor of mPlum or other known far-red fluorescent proteins. This motivated us to explore biosensors utilising homo-FRET readouts of mCherry that can be quantitatively analysed using time-resolved anisotropy.

### 1.2. Fluorescence Anisotropy

Fluorescence anisotropy refers to the degree of polarisation of a population of fluorophores [9]. If a sample is excited with polarised light, the fluorophores whose dipoles are aligned more closely to the excitation polarisation will be more strongly excited and the emitted fluorescence will be partially polarised. The degree of polarisation of the fluorophores will decrease over time after excitation, due to rotational “dephasing” of the fluorophores due to collisions with solvent molecules. This behaviour is described by Equation (1) in which the intensity of light emitted polarised at a particular angle ψ to polarisation of excitation light, *I*_ψ_(*t*), is given by:

(1) $$I_{\text{ψ}}(t)=\frac{I_{t}}{3}\left[1+\left(3\cos{\text{ψ}}^{2}-1\right)\cdot r(t)\right]$$

where *I_t_*(*t*) is the total emission intensity as a function of time [9]. The time-resolved anisotropy parameter *r*(*t*) in Equation (1) may be determined from measurements of the emission intensity polarised parallel and perpendicular to the excitation light according to:

(2) $$r(t)=\frac{I_{∥}(t)-I_{⊥}(t)}{I_{∥}(t)+2I_{⊥}(t)}$$

**Figure 1:**
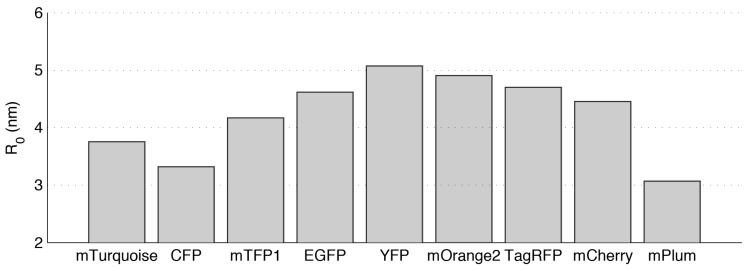
Homo-FRET Förster distance for a number of common fluorophores Plot of the Förster distance *R*_0_ for homo-FRET between common fluorescent proteins calculated as described in the supplementary methods.

The steady state anisotropy, *r_ss_*, can be calculated in an analogous fashion using steady state measurements of $I_{∥}$ and $I_{⊥}$. If the fluorophore population is undergoing homo-FRET, *i.e.*, resonant energy transfer between fluorophores of the same species-the fluorescence anisotropy will decrease as fluorophores can then transfer energy to other fluorophores whose dipoles were far from alignment with the polarisation of the excitation radiation. Thus measurements of time resolved or steady state anisotropy and/or *r*(*t*) can provide a readout of homo-FRET, and past work in this area is reviewed in the next section. Homo-FRET can be used to probe interactions between proteins labelled with spectrally identical fluorophores. Measurements of homo-FRET inherently requiring less spectral bandwidth than hetero-FRET and therefore providing more scope for multiplexing different homo-FRET biosensors with well separated emission spectra. In general, the Förster distances for homo-transfer of energy between fluorescent protein (FP) molecules are comparable to those of hetero-FRET. Figure 1 shows the homo-transfer Förster distances for a range of common FPs. Supplementary Figure S1 shows a table of Förster distances for hetero- and homo-FRET for the same FPs. For mCherry, the Förster distance for homo-transfer between molecules is 4.45 nm, which is comparable to the value of 4.83 nm for hetero-FRET transfer between ECFP and EYFP.

In any experiment the fluorescence intensity decays measured in each polarisation channel will be convolved with the instrument response function and, since convolution does not commute with division, the time-resolved anisotropy parameter *r*(*t*) cannot be accurately determined using Equation (2), particularly if the instrument response functions are not identical for the two channels. Instead it is more accurate to directly fit the measured intensity decays in each polarisation channel to the model described by Equation (1) [10].

Besides FRET, it is likely that in most experiments there will be other processes contributing to time-varying depolarisation of the fluorescence that will be manifest as additional components in the fluorescence anisotropy decay profile. If time and polarisation resolved measurements are made, it may be possible to resolve these different components. In the case of a homo-FRET experiment involving fluorescent proteins, the dominant contributions to the fluorescence anisotropy decay are a slow depolarisation due to rotation of the fluorophore on a scale of tens of nanoseconds, and a fast depolarisation component due to FRET. In this case the time resolved anisotropy parameter may be expressed as:

(3) $$r(t)=r_{\infty }+\sum_{k}r_{k}\exp\left(-\frac{t}{{\text{θ}}_{k}}\right)$$

where $r_{\infty }$ is the limiting anisotropy as t→∞ and θ*_k_* is the characteristic lifetime of the *k*^th^ anisotropy component [9].

### 1.3. Experimental Approaches to Homo-FRET Imaging

A number of previous approaches to homo-FRET have been demonstrated. Qualitative readouts of homo-FRET have been realised using measurements of the steady state anisotropy [11,12,13] where continuous wave excitation and detection provides the time average of Equation (1) for imaging channels with the polarisation resolved parallel and perpendicular to the incident polarisation. However, while steady-state fluorescence anisotropy imaging experiments provide an effective approach to detect and map both hetero-FRET and homo-FRET, they do not provide sufficiently quantitative readouts to enable the complex decays to be fitted to models representing the underlying molecular processes. Time-resolved fluorescence anisotropy imaging can provide further quantitation, e.g., to study protein interactions, particularly oligomerisation as discussed below.

Gautier *et al.* [14] used a confocal TCSPC system with a Fresnel rotator in the excitation path with a fixed analyser in the detection path to sequentially record the emission polarised parallel and perpendicular to the excitation at fixed points. The authors used this system to measure dimerisation of herpes simplex virus thymidine kinase (TK) fused to green fluorescent protein (GFP). By reconstructing the anisotropy decay using Equation (2) and fitting to a bi-exponential model the anisotropy decay components associated with rotational motion and FRET were resolved.

Clayton *et al.* [15] demonstrated a confocal frequency domain TR-FAIM system implemented on a modified frequency domain FLIM microscope where images were acquired consecutively at different polarisation angles. The authors derived analytical expressions for the parameters of a mono-exponential anisotropy decay with a finite limiting anisotropy $r_{\infty }$ in terms of the measured phase and amplitudes. This approach was later extended to wide-field imaging with simultaneous acquisition of the polarisation-resolved images using an image splitter with a wide-field frequency domain microscope system [16]. The author extended the phasor graphical approach to the analysis of time resolved anisotropy data and the system to study dimerisation of epidermal growth factor (EGFR) tagged with EYFP [17].

Bader *et al.* [18] demonstrated a confocal polarisation resolved time gated microscope which was applied to estimate the size of clusters of GPI-GFP, a lipid raft marker. The system employs two time-resolved detection channels (utilising 4 time gates of 2 ns width) to simultaneously capture fluorescence analysed at perpendicular polarisations. The GFP-GPI cluster size was estimated using the limiting anisotropy $r_{\infty }$ and this approach was later applied to a number of different GFP fusions [19]. Thaler *et al.* [20] used polarised resolved TCSPC imaging of Venus-tagged CaMKIIα to investigate dimer formation and regulation of the domain. The authors reconstructed the average anisotropy decay over a number of cells using Equation (2) and fitted globally to a bi-exponential model to determine the rotational correlation times for multimers of different sizes. They then used steady state anisotropy to image dimer formation and separation in live cells.

### 1.4. Quantifying Homo-FRET Aggregation Using Time Resolved Anisotropy

Time resolved measurements of the anisotropy decay may be used to provide information about the clustering parameters of the molecules undergoing FRET. This section will consider the expected anisotropy decay in the presence of homo-FRET between a cluster of identical fluorophores using the approach developed by Runnels and Scarlata [21]. The rate equations for homo-FRET are more involved than those for hetero-FRET because it is possible that multiple FRET transfers steps may occur before emission since there is symmetry between the molecules in the cluster.

A simplified model where the transfer rate is equal between all molecules in the cluster will be considered. While this assumption can only be approximate for clusters of *N* > 3 due to geometrical constraints, we note that Runnels and Scarlata [21] considered the case where the distance between molecules in a cluster is uneven and showed that when the relative distance between all three molecules is less than 0.8*R*_0_, where *R*_0_ is the Förster distance, the distance at which the FRET efficiency is 50%, the emission anisotropy is relatively insensitive to differences in the distance between molecules and is mainly sensitive only to the number of interacting molecules. Thus this assumption is not unreasonable for larger clusters.

For a population of *N* identical, randomly oriented fluorophores with fluorescence lifetime τ in a cluster where one fluorophore is stimulated into the excited state at time *t* = 0, it may be shown that the probability that the initially excited molecule remains in an excited state at time *t* is given by:

(4) $${\text{ρ}}_{1}(t)=\frac{1}{N}\left(1+\left(N-1\right)e^{-Nk_{F}t}\right)e^{-\frac{t}{\text{τ}}}$$

Where *k_F_* is the homo-FRET rate, τ is the fluorescence lifetime and *N*, the number of fluorophores in the cluster is an integer, while the probability that the *i*^th^ molecule is in the excited state is:

(5) $${\text{ρ}}_{i}(t)=\frac{1}{N}\left(1-e^{-Nk_{F}t}\right)e^{-\frac{t}{\text{τ}}}$$

This derivation is shown in the supplementary information. We can then calculate the respective probabilities that a photon is emitted by either the initially excited fluorophore or one of the other fluorophores in the cluster. The probability of a photon being emitted by the initially excited molecule is given by:

(6) $$\begin{array}{ll} p_{1}(t) & =\frac{{\text{ρ}}_{1}(t)}{{\text{ρ}}_{1}(t)+(N-1){\text{ρ}}_{i}(t)}\\ & =\frac{1}{N}\left(1+\left(N-1\right)e^{-Nk_{F}t}\right) \end{array}$$

while the probability of a photon being emitted by one of the remaining *N* − 1 fluorophores is:

(7) $$\begin{array}{ll} p_{i}(t) & =\frac{\left(N-1){\text{ρ}}_{i}(t\right)}{{\text{ρ}}_{1}(t)+(N-1){\text{ρ}}_{i}(t)}\\ & =\frac{1}{N}\left(N-1\right)\;\left(1-e^{-Nk_{F}t}\right) \end{array}$$

Agranovich and Galanin [22] showed that, for such a system, the average anisotropy for emission after a single transfer is *r_et_* = 0.016. If the anisotropy of emission by the initially excited fluorophore at time *t* = 0 is given by *r*_0_ then, if FRET is the only process leading to depolarisation, the time evolution of the anisotropy is given by:

(8) $$\begin{array}{ll} r\left(t\right) & =p_{1}r_{0}+p_{i}r_{et}\\ & =\frac{1}{N}\left[r_{0}\left[1+\left(N-1\right)e^{-Nk_{F}t}\right]+r_{et}\left(N-1\right)\left(1-e^{-Nk_{F}t}\right)\right] \end{array}$$

If the molecules are free to rotate there will be a further decay in the anisotropy due to rotation and, assuming a spherical molecule, the decay can be characterised by a decay lifetime θ*_R_*. Since anisotropies combine multiplicatively [9], the total anisotropy will be:

(9) $$\begin{array}{ll} r\left(t\right) & =\frac{1}{N}\left[r_{0}\left[1+\left(N-1\right)e^{-Nk_{F}t}\right]+r_{et}\left(N-1\right)\left(1-e^{-Nk_{F}t}\right)\right]e^{-\frac{t}{{\text{θ}}_{R}}}\\ & =\frac{\left(N-1\right)}{N}\left(r_{0}-r_{et}\right)e^{-\left(\frac{1}{{\text{θ}}_{R}}+Nk_{F}\right)t}+\frac{1}{N}\left[r_{0}+\left(N-1\right)r_{et}\right]e^{-\frac{t}{{\text{θ}}_{R}}} \end{array}$$

If the data is fitted to a model with two anisotropy decay rates corresponding to the molecular rotational decorrelation (*r*_1_, θ_1_) and the homoFRET (*r*_2_, θ_2_) respectively, then

(10) $$r\left(t\right)=r_{1}\exp\left(-\frac{t}{{\text{θ}}_{1}}\right)+r_{2}\exp\left(-\frac{t}{{\text{θ}}_{2}}\right)$$

where θ_1_ > θ_2_ then, by comparing Equations (9) and (10), the measured parameters will be given by:

(11) $$r_{1}=\frac{1}{N}\left(r_{0}+\left(N-1\right)r_{et}\right)$$

(12) $$r_{2}=\frac{\left(N-1\right)}{N}\left(r_{0}-r_{et}\right)$$

and

(13) $${\text{θ}}_{1}={\text{θ}}_{R}$$

(14) $$\;{\text{θ}}_{2}={\left(\frac{1}{{\text{θ}}_{R}}+Nk_{F}\right)}^{-1}$$

In many cases $Nk_{F}\gg {{\text{θ}}_{R}}^{-1}$ and so it is reasonable to make the approximation: $\;{\text{θ}}_{2}\approx {\left(Nk_{F}\right)}^{-1}$. The rotational correlation time of GFP alone has been reported as 36 ns [23], and it is likely the rotational correlation time of a FP tagged protein will be higher. The FRET transfer rate will of course depend strongly on the biosensor; for a FRET efficiency of 50%, the rate will be equal to the inverse of the donor lifetime, 3–4 ns for many common FPs.

By comparing Equations (9) and (10) at *t* = 0 it can be seen that *r*_1_ + *r*_2_ = *r*_0_, Equations (11) or (12) can then be used to express the number of molecules, *N*, in a cluster in terms of the fitted parameters:

(15) $$N=\frac{r_{1}-r_{et}+r_{2}}{r_{1}-r_{et}}=1+\frac{r_{2}}{r_{1}-r_{et}}$$

If a pixel contains a mixture of *N*-mers and monomers the experimentally determined value of *N* given by Equation (15) will in general be a real number and represent a weighted average of the cluster sizes present. If the true integer value of *N* is known or can be estimated, for example in the case of a biosensor, the population fraction of *N*-mers β and a population fraction of monomers 1 − β may be estimated. For such a mixture, the average time resolved anisotropy is given by:

(16) $$r\left(t\right)=\text{β}\frac{\left(N-1\right)}{N}\left(r_{0}-r_{et}\right)e^{-\left(\frac{1}{{\text{θ}}_{R}}+Nk_{F}\right)t}+\left[r_{0}-\text{β}\frac{\left(N-1\right)}{N}\left(r_{0}-r_{et}\right)\right]e^{-\frac{t}{{\text{θ}}_{R}}}$$

and the fitted parameters will be given by:

(17) $$r_{2}=\text{β}\frac{\left(N-1\right)}{N}\left(r_{0}-r_{et}\right)$$

(18) $$r_{1}=r_{0}-\text{β}\frac{\left(N-1\right)}{N}\left(r_{0}-r_{et}\right)$$

thus, β can be calculated using:

(19) $$\text{β}=\frac{N}{N-1}\left(\frac{r_{2}}{r_{2}+r_{1}-r_{et}}\right)$$

In the steady state case the expression for the steady state anisotropy is [21]:

(20) $$r_{ss}=r_{1}\cdot \frac{1+k_{F}\text{τ}}{1+Nk_{F}\text{τ}}+r_{et}\cdot \frac{\left(N-1\right)\cdot k_{F}\text{τ}}{1+Nk_{F}\text{τ}}$$

Therefore the cluster size can only be determined from a steady state measurement if an independent measurement of the transfer rate and the fluorophore lifetime can be made and a theoretical value for *r*_1_ is assumed.

### 1.5. Phosphoinositide and Calcium Signalling

Phosphoinositides, phosphorylated forms of phosphatidylinositol (PtdIns) are membrane-bound lipids that play an important role in signalling in a number of cellular processes, including membrane trafficking, cytoskeletal regulation and chemotaxis [24,25]. The phosphoinositide PtdIns(3,4,5)*P*_3_ plays a key role in a positive feedback mechanism controlling cytoskeleton dynamics [26,27]. PtdIns(3,4,5)*P*_3_ is produced by type I Phosphatidylinositide 3-kinases (PI3Ks) at the plasma membrane and degraded by Phosphatase and tensin homolog (PTEN). PtdIns(3,4,5)*P*_3_ plays an important role in the localisation of cytoskeleton formation and polarised distributions of PtdIns(3,4,5)*P*_3_ are found at the leading edges of a number of cell systems [28,29]. This polarisation has been attributed to PI3K activity at the leading edge and PTEN activation at the trailing edge [30,31]. PtdIns(3,4,5)*P*_3_ promote to the plasma membrane—And thereby activate—A number of key regulators of forward cell motility, including Rho GTPases such as Rac and Cdc42 [32] and Akt [33]. PI3Ks are activated, leading to PtdIns(3,4,5)*P*_3_ accumulation, by a number of receptor tyrosine kinases [34] such as platelet-derived growth factor receptor (PDGFR) a cell surface receptor that is phosphorylated upon binding platelet-derived growth factor (PDGF) [35].

PDGFR also activities an isoform of Phospholipase C (PLC), PLCγ, through interactions with the SH2 domain of PLCγ [36]. PLC catalyses the hydrolysis of the phosphoinositide PtdIns(4,5)*P*_2_ to the second messengers Ins(1,4,5)*P*_3_ and DAG. Ins(1,4,5)*P*_3_ stimulations the release of calcium from intracellular stores [37]. It has been reported that PLC plays a role in sensing direction in growth factor inducted chemotaxis in a number of cell systems [38,39,40]. In particular, PLCγ was shown to be required for chemotaxis of fibroblasts towards PDGF-BB and calcium dynamics modulated by Ins(1,4,5)*P*_3_ were suggested as the primary mechanism for the PLCγ involvement.

Studies have shown that a number of signalling pathways, including events leading to local accumulation of PI(3,4,5)*P*_3_ and calcium signalling, act in parallel to mediate chemotaxis [41,42]. The ability to image multiple signalling components in the same cell may provide a useful tool to elucidate the relationship between these signalling pathways in mediating chemotaxis. The accumulation of 3′ phosphorylated phosphoinositides PtdIns(3,4,5)*P*_3_ and PtdIns(3,4)*P*_2_ (3′ PtdIns) may be monitored using fluorescent protein fusing of the pleckstrin homology domain of Akt (Akt-PH) which bind to these lipids. Previous experiments have demonstrated that AKT-PH fusions may be used to monitor accumulation of this signalling molecule in the plasma membrane [28] by translocation. However, quantification of intensity based readouts are problematic as changes in membrane shape, e.g., due to ruffling, can lead to changes in intensity independent of translocation [43]. FRET has previously been used to report on phosphoinositide translocation and it has been suggested that PtdIns(3,4,5)*P*_3_ localises in microdomains within the plasma membrane [44,45] where the local density may be high enough to allow efficient FRET between bound PH-domains. This approach has previously been demonstrated with both hetero- [46] and homo-FRET [47]. Here we report on our demonstration of TR-FAIM of mCherry-Akt-PH as a quantitative homo-FRET readout for PtdIns(3,4,5)*P*_3_ and PtdIns(3,4)*P*_2_ accumulation and illustrate how this readout can be multiplexed with an ECFP-YFP FRET biosensor for calcium (TN-L15).

## 2. Results and Discussion

### 2.1. Homo-FRET between Aggregating mCherry-Akt-PH Molecules

**Figure 2:**
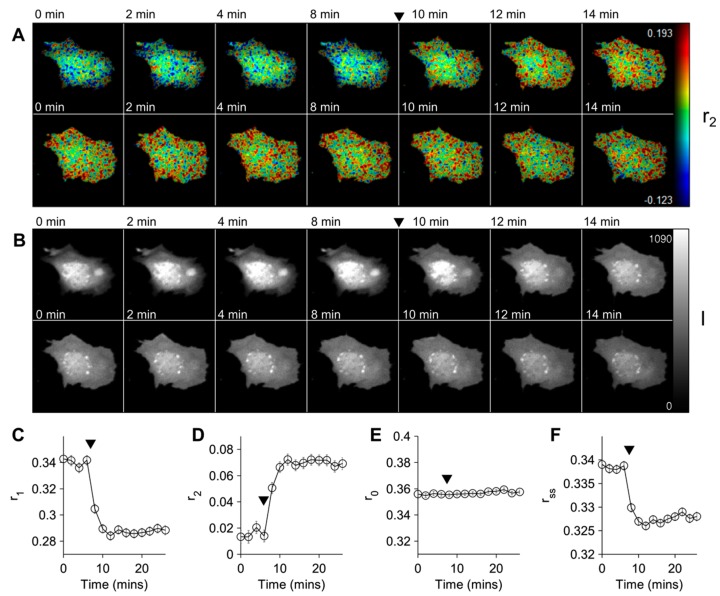
Homo-FRET anisotropy response of mCherry-Akt-PH to PDGF-BB stimulation. A Mouse embryonic fibroblasts (MEF) transfected with mCherry-Akt-PH was imaged on the polarisation resolved microscope at two minute intervals and stimulated with 50 ng/mL PDGF-BB after 8 min (indicated by black triangles). The data was fitted to bi-exponential anisotropy decay model. (**A**) Intensity merged false colour map of the anisotropy component associated with FRET *r*_2_; (**B**) Integrated intensity image; (**C**–**F**) Values of (**C**) anisotropy component due to rotational correlation time *r*_1_; (**D**) anisotropy component associated with FRET *r*_2_, (**E**) initial anisotropy *r*_0_ and (**F**) steady-state anisotropy *r_ss_* averaged across each image. Error bars represent 95% confidence limits across the image, adjusted for smoothing. The cell shown is representative of a series of 10 experiments, of which 7 cells showed a similar response.

In this section the use of mCherry-Akt-PH as a homo-FRET reporter for PtdIns(3,4,5)*P*_3_ and PtdIns(3,4)*P*_2_ accumulation is discussed. Mouse embryonic fibroblasts (MEF) expressing mCherry-Akt-PH were imaged using the TR-FAIM microscope discussed below in Section 3. Images were acquired at two-minute intervals for thirty minutes with a 60 s integration time. After eight minutes the cells were stimulated with 50 ng/mL PDGF. An exemplar dataset is shown in Figure 2. The data was fitted globally assuming a globally constant bi-exponential fluorescence intensity decay profile for mCherry, two globally constant anisotropy correlation times associated with molecular rotation and FRET, and with the initial anisotropy contributions of these two components fitted pixelwise, discussed further in Section 3.3.1. The fitted values of the intensity lifetimes were τ_1_ = 1.62 ns and τ_2_ = 0.816 ns respectively. The fitted contribution of the first component was β = 0.577. The fitted values of the anisotropy correlation times associated with rotation and FRET were θ_1_ = 35.2 ns and θ_2_ = 3.59 ns, respectively. Within two minutes of the PDGF stimulation an increase in the FRET associated anisotropy component *r*_2_ and a corresponding reduction in the rotational component *r*_1_, are observed. The total initial anisotropy *r*_0_ = *r*_1_ + *r*_2_, which is not constrained in the fitting, appears to remain unchanged as expected, indicating the model adequately describes the data.

**Figure 3:**
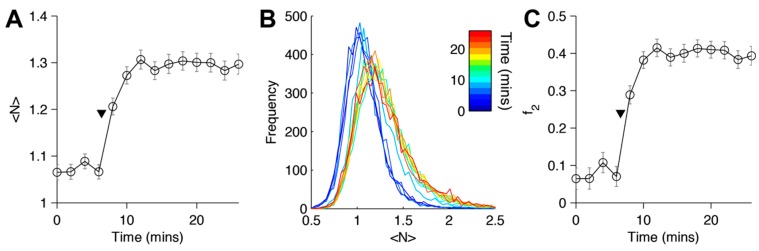
Average values of calculated cluster parameters for a time course of a cell transfected with mCherry-Akt-PH and stimulated with platelet-derived growth factor (PDGF). Cluster parameters for the mCherry-Akt-PH transfected cells shown in Figure 2 as a function of time. (**A**) Average cluster size 〈N〉; (**B**) histogram of average cluster size 〈N〉 at each timepoint and (**C**) average fraction of dimers to monomers, *f*_2_. Error bars represent 95% confidence intervals.

The average cluster size 〈N〉 was calculated on a pixel-by-pixel basis according to Equation (15). Figure 3A,B show 〈N〉 averaged across each image and the histogram of 〈N〉 in each image for one representative TR-FAIM time-course. Both before and after stimulation 〈N〉 is significantly less than 2 and the spread of *N* lies in the range 1–2 within the expected standard deviation of the measurement. Therefore it is reasonable to assume that there is not a significant population of clusters with *N* > 2. The ratio of monomers to dimers, which gives an indication of the ratio of free to membrane bound mCherry-Akt-PH (*f*_2_), was calculated and is shown Figure 3C.

In the derivation of the relationship between the parameters of a fit to two rotational correlation times and clustering parameters we assumed that all the mCherry molecules transferred energy with the same FRET efficiency. Recent studies suggest that the approximation of the decay of a population of fluorescent proteins undergoing FRET with a single efficiency may be insufficient. For example, Vogel *et al.* [48] recently demonstrated that a short Cerulean-YFP linked construct presented a bi-exponential fluorescence decay profile. They proposed that this could be due to lack of rotational mobility of the construct on the time-scale of the fluorescence decay, resulting in a distribution of FRET interactions due to the variety of chromophore angles and distances present in a population of molecules, leading to an apparent distribution of FRET efficiencies. In our experiment this would impact the accuracy of the estimated membrane bound fraction. However, some assumption concerning the FRET efficiency is inevitable and we believe that our calculated readout does correlate with the underlying cell signalling processes and still could provide useful information, for example as a quantitative readout for assays of PtdIns(3,4,5)*P*3 and PtdIns(3,4)*P*2 accumulation.

### 2.2. Multiplexed mCherry-Akt-PH and TN-L15 Measurements

Mouse embryonic fibroblasts (MEFs) were transfected with equimolar concentrations of the FRET biosensors TNL15 and mCherry-Akt-PH. Images were acquired in alternate spectral channels (corresponding to mCherry and ECFP) at 30 s intervals for 30 min. The sample was stimulated with 50 ng/mL PDGF-BB after eight frames.

**Figure 4:**
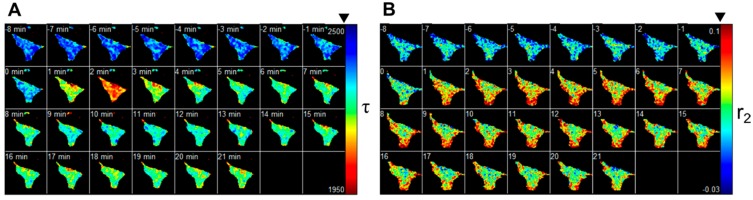

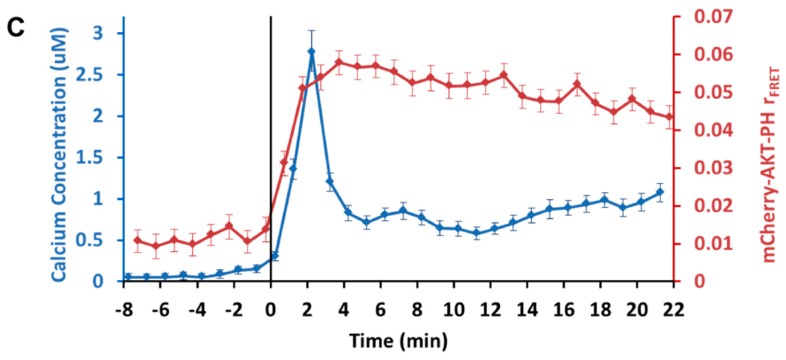
Multiplexed measurement of 3′ PtdIns accumulation and calcium concentration in response to PDGF-BB stimulation in a fibroblast. A MEF expressing TN-L15 and mCherry-Akt-PH was imaged on the polarised resolved microscope and stimulated with PDGF-BB after eight minutes. (**A**) Fitted false colour lifetime map of TN-L15 measuring calcium concentration (**B**) Fitted anisotropy component associated with FRET of mCherry-Akt-PH measuring concentration of 3′ PtdIns; (**C**) Plot of calculated calcium concentration and mCherry-Akt-PH FRET associated anisotropy component. The cell shown is representative of a series of 5 experiments, of which 5 cells showed a similar response.

The TN-L15 polarisation-independent fluorescence intensity decays were reconstructed taking into account the different instrument response functions of the two channels as described the supplementary information. The data was fitted to a mono-exponential decay model on a pixel by pixel basis and the average calcium concentration was estimated using the calibration measured by Laine *et al.* [49]. The response for a representative cell is shown in Figure 4A shows the TNL-15 biosensor lifetime map at each time point and Figure 4B shows the anisotropy component associated with FRET, *r*_2_, for the mCherry-Akt-PH biosensor. Figure 4C shows the average estimated calcium concentration and the estimated fraction of mCherry-Akt-PH dimers averaged across the cell with time. Both the levels of 3′ PtdIns and calcium concentration increased within a minute of stimulation. The calcium concentration peaks approximately two minutes after stimulation and rapidly equilibrates at a level above that of the pre-stimulation level. After stimulation there appear to be higher levels of calcium at the membrane regions-in contrast to the uniform calcium concentration pre-stimulation. The level of 3′ PtdIns peaks more slowly at around four minutes and appears to drop more gradually. However it should be noted that the low dissociation constant of Akt-PH may artificially maintain the 3′ PtdIns concentration in the membrane by blocking phosphatase binding. We note that, as with most multiplexed FRET experiments, we rely on the fact that the two biosensors are not co-localised within the Förster distance and so we do not expect inter-molecular FRET between the two biosensors. The TN-L15 calcium sensor predominately localises at the cytoplasm. The characteristic fluorophore concentration at which FRET occurs between homogeneous solutions of fluorophores is given by C0=(43πR03)−1 [9]; for the Förster distance for FRET between CFP and mCherry, 4.64 nm, the characteristic fluorophore concentration is 4 mM. This is several orders of magnitude higher than the level of overexpressed PH domain fusion constructs cells are able to tolerate [46] and thus no significant inter-molecular FRET would be expected. If two biosensors were colocalised within small microdomains, e.g., in the same protein clusters, it is possible FRET would occur between the CFP and YFP with mCherry. This would lead to an artefactual reduction in the lifetime of the shorter wavelength pair.

## 3. Experimental Section

### 3.1. Cells and Plasmids

Immortalized wild type (WT) and PLCε null allele (KO) mouse embryonic fibroblasts (MEFs) were generated as described in [50] were cultured in 4.5 g/L glucose DMEM medium supplemented with 10% FBS, 2 mM l-glutamine and antibiotics (50 U/mL penicillin, 50 μg/mL streptomycin) at 37 °C and 5% CO_2_. MEFs were transfected by electroporation using the Amaxa Nucleofector II (Lonza, Basel, Switzerland) using Ingenio electroporation solution (Mirus, Madison, WI, USA) with program A-23 and 2 µg of plasmid DNA. The transfected cells were transferred to 35 mm glass bottom dishes (Matek, Ashland, MA, USA) containing 1 mL of growth media. The cells were allowed to recover for two hours, then starved in DMEM supplemented with 0.5% FBS overnight. The cells were imaged in Earle’s Balanced Salt Solution (EBSS) supplemented with 4 mM glutamine, 1 mM sodium pyruvate and 2 mM CaCl_2_. The cells were stimulated with 50 ng/mL PDGF-BB (R&D Systems, Minneapolis, MN, USA)

GFP–Akt-PH domain plasmid was generated by using a PCR fragment of bovine PKB/Akt encoding the N-terminus (residues 1–147) and sub-cloning into the pmCherry-C1 vector. The plasmid coding for TN-L15 was a kind gift of Dr. O. Grisbeck (Max-Planck-Institut für Neurobiologie, Martinsried, Germany) [5].

### 3.2. Imaging

**Figure 5:**
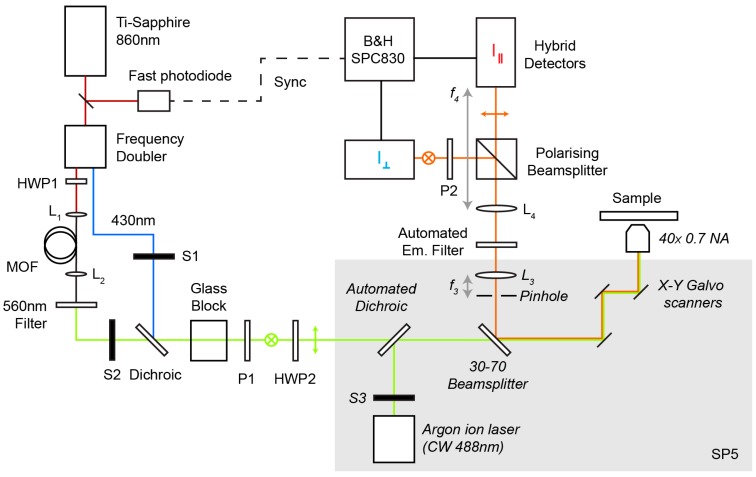
Schematic of the multiplexed polarisation resolved microscope described in the text. Components inside the grey area and labelled in italics are internal to the SP5 confocal microscope. Abbreviations used: *HWP*, half wave plate; *S*, shutter, *P*, polariser, *L*, lens. The focal lengths of lenses *L*_3_ and *L*_3_ are labelled *f*_3_ and *f*_4_, respectively.

Imaging was performed on a modified commercial confocal laser scanning microscope (SP5, Leica Microsystems, Wetzlar, Germany, SP5), illustrated schematically in Figure 5. For excitation the system uses a femtosecond mode-locked Ti:Sapphire laser (MaiTai, Spectra Physics, Santa Clara, CA, USA) with an repetition rate of 80 MHz was tuned to 860 nm. To excite the ECFP donor of the TN-L15 biosensor the Ti:Sapphire beam was frequency doubled (Inspire Blue, Spectra Physics) to 430 nm. The 430 nm beam was passed through a glass block to stretch the temporal width of the excitation pulses to picoseconds to prevent excessive phototoxicity [51,52] and photobleaching due to nonlinear effects. A micro-structured optical fibre (MOF) (PM-750, NKT Photonics, Birkerød, Denmark) was pumped by the Ti:Sapphire to generate a supercontinuum. An optical isolator was used to prevent back reflections from the input face of the MOF into laser cavity. The supercontinuum source was used to excite mCherry with a 560/40 nm filter (D560/40 m, Chroma, Bellows Falls, VT, USA). The frequency doubled and supercontinuum excitation beams were independently shuttered and combined using a 450 nm long pass dichroic (T455LP, Chroma). These collimated beams were polarised using a polarising beam splitter (P1) and the final polarisation angle adjusted using a half wave plate (HWP2).

The de-scanned collimated emission light was passed through an automated filter wheel to select the desired emission wavelength and divided into two detection channels using a polarising beam splitter cube and two hybrid photomultipliers (HPM-100-40, Becker and Hickl, GmbH, Berlin, Germany). A bleed-through from the parallel channel into the perpendicular channel of approximately 3% was observed after the beam splitter and so a wire grid polariser (P2) was inserted before the perpendicular detector, resulting in an overall system extinction ratio >150. The fluorescence signals were measured using TCSPC implemented using a SPC830 module (Becker and Hickl, GmbH). A trigger signal was generated by a fast photodiode (DET10C, Thor Labs, Newton, NJ, USA) with a beam picked off from the Ti:Sapphire output. A computer controlled delay line (Kentech Instruments Ltd., Oxford, UK) was used to control the timing of the trigger signal relative to the detected signal.

The system was automated using Micromanager [53], an open source framework for controlling microscope systems that provides adaptors for a wide range of devices including the filter wheels and shutters. A C++ driver was written to control the delay box to compensate for the difference in path length between the frequency doubled and MOF-generated excitation pulses. A 40× 0.75 NA objective was used for imaging with the confocal pinhole set to one Airy unit. Data was collected in 256 time-bins over a measurement period of 12.5 ns. 128 × 128 pixel images were acquired sequentially in each channel with a line scan rate of 400 Hz. Frames in each channel were accumulated for either 30 s acquisition (Figure 2) or 60 s (Figure 4), giving a total pixel integration time of 29.3 or 58.6 µs, respectively, per image.

### 3.3. Instrument Response Function (IRF) Measurement and Polarisation Alignment

Quantitative analysis of FLIM and TR-FAIM data requires knowledge of the instrument response function (IRF) and this was measured using the excitation light back scattered from a diffuse sample by inserting an OD 3.0 neutral density filter in the excitation path and rotating the polarisation of the excitation beam using HWP1 until the peak intensity was similar in both channels. The reference measurements in both channels were independently normalised to unity.

Again using a diffuse back-scattering sample, the polarisation of the excitation beam was then aligned with the parallel detector channel by rotating HWP1 until the signal in the perpendicular channel was minimised. An extinction ratio greater than 100 was considered acceptable. To determine the relative detection efficiency (*g* factor) of the two channels, which depend on polarisation effects in the microscope and detection path optics, a reference dye measurement was made with the same filter set as was used in the cell imaging experiments. Fluorescein and Rhodamine 6G were used for the ECFP and mCherry spectral channels, respectively. Both dyes have a rotational correlation time of θ ≈ 200 ps and a mono-exponential decay profile. After t ~ 5θ the dye is completely depolarised and so the emitted intensity is equal in each polarisation. The ratio of the dye fluorescence measured in each channel at t > 5θ was used to determine the *g* factor.

### 3.4. Analysis

#### 3.4.1. Fitting of Anisotropy Data

Anisotropy data was fitted using global analysis in FLIMfit [47]. A model with a bi-exponential fluorescence intensity decay, with lifetimes τ_1_ and τ_2_, relative contribution of the first component of β_1_, and two rotational correlation times, θ_1_ and θ_2_ with respective initial anisotropy contributions *r*_1_ and *r*_2_, was used. The polarisation independent total intensity *I_T_*(*t*) and the anisotropy *r*(*t*, ψ) therefore took the form:

(21) $$I(t)=I_{0}\left({\text{β}}_{1}\exp\left(-\frac{t}{{\text{τ}}_{1}}\right)+\left(1-{\text{β}}_{1}\right)\exp\left(-\frac{t}{{\text{τ}}_{2}}\right)\right)$$

(22) $$r(t,\text{ψ})=r_{1}\exp\left(-\frac{t}{{\text{θ}}_{1}}\right)+r_{2}\exp\left(-\frac{t}{{\text{θ}}_{2}}\right)$$

The complete model fitted to the data, before convolution and accounting for incomplete decays, can be obtained by inserting Equations (21) and (22) into Equation (1), giving:

(23) $$I(t,\text{ψ})=\frac{I_{0}}{3}\underset{A}{\underset{︸}{\left({\text{β}}_{1}\exp\left(-\frac{t}{{\text{τ}}_{1}}\right)+\left(1-{\text{β}}_{1}\right)\exp\left(-\frac{t}{{\text{τ}}_{2}}\right)\right)}}\cdot \underset{B}{\underset{︸}{\left[1+\text{η(ψ})\left(r_{1}\exp\left(-\frac{t}{{\text{θ}}_{1}}\right)+r_{2}\exp\left(-\frac{t}{{\text{θ}}_{2}}\right)\right)\right]}}$$

The first portion (A) corresponds to the bi-exponential intensity decay of mCherry while the second portion is the polarisation dependent bi-exponential anisotropy decay discussed in Section 1.4. $\text{η(ψ})=3\cos{\text{ψ}}^{2}-1$ is a factor which accounts for the differences between the intensity measured in the parallel (ψ = 0) and perpendicular (ψ = 90°) channels.

The lifetimes, lifetime contributions and anisotropy decay times were treated as global variables, *i.e.*, invariant (but unknown) across the dataset, while the relative contributions of the anisotropy components *r*_1_ and *r*_2_ were treated as local variables and so could vary from pixel to pixel. To test whether it is appropriate to assume a constant fluorescence intensity decay profile during the fitting, the intensity decays were reconstructed as described in the supplementary methods and fitted on a pixel-by-pixel basis to a mono-exponential model. The variation in mean lifetime across the images time-series was found to be less than 25 ps.

#### 3.4.2. Calibration of Calcium Measurements

To estimate the calcium concentration based on the measured TN-L15 decay profiles the methodology described by Laine *et al.* [49] was used. Laine measured the lifetime response of a cytosol extract of the TN-L15 biosensor at different calcium concentrations. The solutions were maintained at 37 °C and pH 7.2 to mimic the conditions found in cells. The measured decay profiles were fitted to mono-exponential decay model to determine the mean lifetime.

Using this data, a calibration curve was calculated to convert between the measured lifetime of TN-L15 and calcium concentration. The data were fitted to a Hill-Langmuir sigmoid [54]:

(24) $$\;\text{τ}=\left({\text{τ}}_{\text{min}}-{\text{τ}}_{\text{max}}\right)\frac{\left[{\text{Ca}}^{\text{2+}}\right]}{k_{D}+\left[{\text{Ca}}^{\text{2+}}\right]}+{\text{τ}}_{\text{min}}$$

using a non-linear least squares approach to determine the unknown parameters *k*_D_, the dissociation constant and τ_min_, τ_max_, the limits of the lifetime as $\left[{\text{Ca}}^{\text{2+}}\right]\to 0$ and $\left[{\text{Ca}}^{\text{2+}}\right]\to \infty$. The calcium concentration was then calculated based on the measured lifetime using:

(25) $$\left[{\text{Ca}}^{2+}\right]=k_{d}\frac{{\text{τ}}^{\prime }}{1-{\text{τ}}^{\prime }},\;\;\;{\text{τ}}^{\prime }=\frac{\text{τ}-{\text{τ}}_{\min}}{{\text{τ}}_{\max}-{\text{τ}}_{\min}}$$

The excitation and emission wavelengths were matched between the calibration experiments.

#### 3.4.3. Reconstruction and Fitting of Enhanced Cyan Fluorescent Protein (ECFP) Intensity Decays

ECFP intensity decay profiles for the TN-L15 biosensor were reconstructed from the polarisation-resolved data as described in the supplementary methods. The reconstructed TN-L15 ECFP decay profiles were fitted to a mono-exponential decay model on a pixel-by-pixel basis using a maximum likelihood estimator [55]. While ECFP is well known to exhibit a complex decay profile, fitting to a mono-exponential decay is sufficient to determine the mean lifetime of the biosensor. 3 × 3 smoothing was applied to the image data. The calibration curve was used to convert the measured lifetimes to calcium concentrations.

## 4. Conclusions

We developed a model for the time resolved anisotropy decay of a cluster of molecules undergoing homo-FRET was presented, relating cluster size and population fraction parameters to the parameters of a bi-exponential fit to the anisotropy decay profile. By applying global analysis, this model was used to quantify the clustering parameters of a mCherry homo-FRET readout for PtdIns(3,4,5)*P*_3_ and PtdIns(3,4)*P*_2_ accumulation. We note that the quantitative information concerning clustering parameters obtained from the time resolved anisotropy readout of mCherry-Akt-PH would not be possible with a steady state measurement without auxiliary time resolved experiments to determine the fluorescence lifetime. Homo-FRET readouts of mCherry-Akt-PH are more robust than previously reported Hetero-FRET biosensors in the red spectral region owing to mCherry’s superior performance in terms of photosensitisation and photodamage; we demonstrate live cell imaging over a 30 min time course with no significant reduction in fluorescence intensity.

This homo-FRET readout of the mCherry 3′ PtdIns reporter was multiplexed with a conventional ECFP-YFP hetero-FRET calcium biosensor (TNL-15). We believe that this indicates the potential of quantitative time-resolved homo-FRET readouts of biosensors and their scope for multiplexed measurements of cells signalling networks. The use of homo-FRET readout is not limited to dimerisation and aggregation studies; conventional intramolecular GFP-mCherry hetero-FRET sensors could be converted to mCherry homo-FRET sensors by replacing the GFP donor with mCherry, potentially enabling a broad range of multiplexing experiments with relatively minor modifications to existing biosensors.

We note that many biological responses are more rapid than the timescales probed here (where the multiplexing experiment represented in Figure 4 was performed at 1 min intervals with 30 s acquisition per channel) and that such fast biological processes may not be adequately samples by time-resolved laser scanning microscopy TCSPC measurements, which are inherently limited in speed by TCSPC electronics and power constraints due to photobleaching and phototoxicity. However both the signalling pathways imaged in this experiment, which are downstream of the more rapid RTK response, are sufficiently slow that the rising and falling edges of the stimulation response are spread over more than two time points. Faster signalling events could be studied using the approach presented in this paper adapted to more rapid wide-field FLIM instrumentation utilising time-gated or frequency-modulated detection, for which images may be acquired in a few seconds, depending on the sample brightness and photostability.

## Supplementary Materials

Supplementary materials can be found at http://www.mdpi.com/1422-0067/16/07/14695/s1.
