# Supplementary material for: Homo-FRET Based Biosensors and Their Application to Multiplexed Imaging of Signalling Events in Live Cells
Source: Int J Mol Sci. 2015 Jun 30;16(7):14695–716. doi: 10.3390/ijms160714695 (PMC4519867; doi:10.3390/ijms160714695)
Supplement: Supplementary file 1 [file ijms-16-14695-s001.pdf]

Supplementary Information

S1. Calculation of Förster Radius for Homo-Transfer between Common Fluorescent Proteins

The Förster distance R_0, defined as the separation at which FRET activity accounts for half of the decays from the excited state, given by [9]

$$R_0^6 = Q_D \kappa^2 \frac{9000 \ln(10)}{128\pi^5 N_A n^4} J$$
$$J = \int q_d(\lambda) \epsilon_a(\lambda) \lambda^4 d\lambda \tag{1}$$

where Q_D is the fluorescence quantum yield of the donor molecule, n the refractive index of the medium and N_A Avogadro's number. κ^2 is the dipole orientation factor which gives a measure of the relative orientation of the donor and acceptor molecules dipole moments. The overlap integral, J, gives a measure of the overlap between the donor and acceptor spectra, $q_d(\lambda)$ is the normalised donor emission intensity as a function of wavelength and $\epsilon_a(\lambda)$ the extinction coefficient of the acceptor as a function of wavelength. The Förster distances shown in Figures 1 and S1 were calculated using Equation (1) using the photophysical properties of the fluorescent proteins collated by the Tsien lab [56]. The overlap integration J was calculated numerically using the fluorescent protein extinction spectra ($\epsilon_a(\lambda)$) and emission spectra ($q_d(\lambda)$) published on the Tsien lab website [57] measured at 1 nm intervals. The quantum efficiency (QD) of the fluorescent proteins was taken from [56]. The refractive index n was taken to be 1.33, the refractive index of water. The dipole orientation factor was assumed to take a value of $\kappa^2 = 2/3$, the value found in the dynamic averaging case [9], enabling the relative distances to be compared.

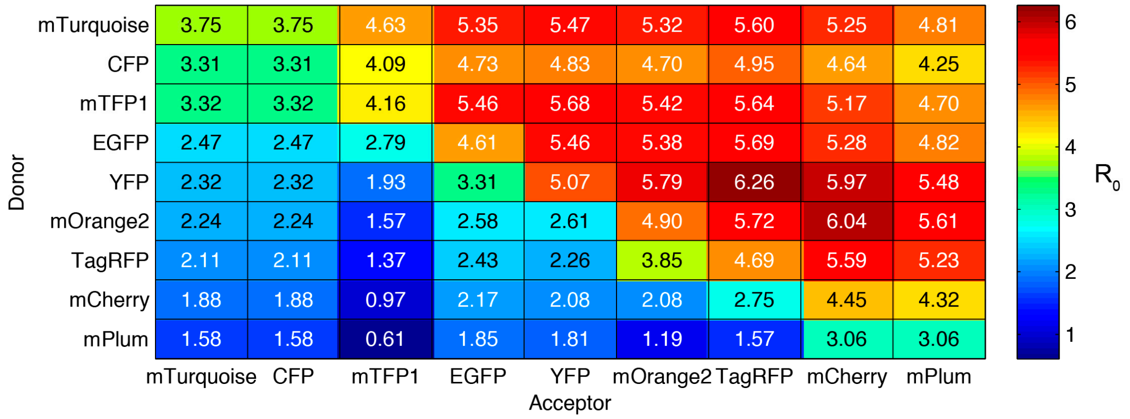

Figure S1. Förster distances for pairs of common fluorescent proteins. Theoretical Förster distance R_0 calculated based on published excitation and emission spectra, extinction coefficients and quantum yields.

S2. Reconstruction of (Polarisation-Independent) Intensity Decay from Polarisation Resolved Data

It is sometimes desirable to reconstruct the polarisation independent decay $I_t(t)$ from the polarisation resolved measurements. Using Equation (1) it is clear, neglecting the effect of the instrument response function, that

$$I_t(t) = I_{\parallel}(t) + 2 \cdot I_{\perp}(t) \tag{2}$$

If the IRF is identical for both channels then Equation (2) may be used since convolution is distributive. Since two different detectors are used for the polarisation channels, however, Equation (2) does not apply. In particular there is a path length difference between the two detectors and the sample, leading to a time delay between the channels.

We may take advantage of the commutivity of convolution (denoted by $\otimes$) to obtain the decay from each channel convolved with the combined IRF, $g_{\perp} \otimes g_{\parallel}$ by computationally convolving each channel with the IRF from the other channel (*i.e.*, $g_{\perp}$ or $g_{\parallel}$). The convolution was performed using the MATLAB function. The polarisation independent decay, convolved with the combined IRF, $\tilde{I}_t(t)$, may then be obtained using Equation (2) and the re-convolved decays as follows

$$\begin{aligned}\tilde{I}_t(t) &= g_{\perp}(t) * \tilde{I}_{\parallel}(t) + 2 \cdot g_{\parallel}(t) * \tilde{I}_{\perp}(t) \\ &= g_{\perp}(t) * \left(g_{\parallel}(t) * I_{\parallel}(t)\right) + 2 \cdot g_{\parallel}(t) * \left(g_{\perp} * I_{\perp}(t)\right) \\ &= g_{\perp}(t) * g_{\parallel}(t) * I_t(t)\end{aligned} \tag{3}$$

S3. Time Evolution of Excitation States in a Cluster

Consider a population of N identical, randomly oriented fluorophores with fluorescence lifetime τ in a cluster where one fluorophore is stimulated into the excited state at time $t = 0$. We wish to determine the probability that the initially excited fluorophore is in the excited state, $\rho_1(t)$ and the probability that one of the remaining $N-1$ fluorophores is in the excited state, $\rho_i(t)$.

The rate of change of the probability that the initially excited fluorophore is in the excited state, $\rho_1(t)$, is determined by three processes, (A) the rate at which the fluorophore decays via non-FRET radiative or non-radiative processes; (B) the rate at which FRET occurs from the initially excited fluorophore to other $N-1$ fluorophores in the cluster and (C) the rate at which FRET occurs from the other fluorophores in the cluster to the initially excited fluorophore. If the lifetime of the fluorophores in the absence of FRET is τ and the rate constant of FRET between any two fluorophores in the cluster, assumed to be constant (*i.e.*, the separation distance is equal between all fluorophores) is k_F, then the rate of change is given by

$$\begin{aligned}\frac{\partial \rho_1(t)}{\partial t} &= \underbrace{-\frac{\rho_1}{\tau}}_{A} \underbrace{- k_F(N-1)\rho_1}_{B} + \underbrace{k_F(N-1)\rho_i}_{C}, \\ &= -\frac{\rho_1}{\tau} + k_F(N-1)(\rho_i - \rho_1)\end{aligned} \tag{4}$$

The rate of change of the probability that one of the remaining $N-1$ fluorophores is in the excited state, $\rho_i(t)$, is determined by four processes; (A) the rate at which the fluorophore decays via non-FRET radiative or non-radiative processes; (B) the rate at which FRET occurs from the other $N-1$ fluorophores in the cluster to the fluorophore; (C) the rate at which FRET occurs from the fluorophore to the initially excited fluorophore and (D) the rate at which FRET occurs from the fluorophore to the other $N-2$ fluorophores in the rest of the cluster.

$$\begin{aligned}\frac{\partial \rho_i(t)}{\partial t} &= \underbrace{-\frac{\rho_i}{\tau}}_{A} \underbrace{-k_F(N-1)\rho_i}_{B} + \underbrace{k_F\rho_1}_{C} + \underbrace{k_F(N-2)\rho_i}_{D} \\ &= -\frac{\rho_i}{\tau} + k_F(\rho_1 - \rho_i).\end{aligned} \tag{5}$$

The boundary conditions for the rate equations are given by

$$\begin{aligned}\rho_i(t=0) &= \begin{cases}1, & i=1 \\ 0, & i>1\end{cases}, \\ \rho_i(t=\infty) &= 0,\end{aligned} \tag{6}$$

Assume that a solution exists of the form

$$\rho_1 = \left(A + Be^{-\alpha t}\right)e^{-\frac{t}{\tau}}, \tag{7}$$

$$\rho_i = \left(C + De^{-\alpha t}\right)e^{-\frac{t}{\tau}}. \tag{8}$$

Using the initial conditions given by Equation (6), by inspection it may be seen that $A+B=1$ and $C+D=0$ so that

$$\rho_1 = \left(A + (1-A)e^{-\alpha t}\right)e^{-\frac{t}{\tau}}. \tag{9}$$

$$\rho_i = C\left(1 - e^{-\alpha t}\right)e^{-\frac{t}{\tau}}. \tag{10}$$

Substituting Equations (9) and (10) into Equation (4) gives

$$-\frac{A}{\tau} - \left(\frac{1}{\tau} + \alpha\right)(1-A)e^{-\alpha t} = -\left[\frac{1}{\tau} + k_F(N-1)\right]\left(A + (1-A)e^{-\alpha t}\right) + FC(k_F - 1)\left(1 - e^{-\alpha t}\right) \tag{11}$$

Considering Equation (11) in the limit $t \to \infty$ gives

$$-\frac{A}{\tau} = -\left[\frac{1}{\tau} + k_F(N-1)\right]A + k_F C(N-1).\ A = C \tag{12}$$

Comparing terms which are multiples of $e^{-\alpha t}$ in Equation (11) and using Equation (12) gives

$$-\left(\frac{1}{\tau} + \alpha\right)(1-A) = -\left[\frac{1}{\tau} + k_F(N-1)\right](1-A) - k_F A(N-1)\ (1-A)\alpha = k_F(N-1) \tag{13}$$

Inserting Equations (7), (8) and (12) into Equation (5) gives

$$A\left[-\frac{1}{\tau}+\left(\frac{1}{\tau}+\alpha\right)e^{-\alpha t}\right]=-A\left(\frac{1}{\tau}+k_F\right)\left(1-e^{-\alpha t}\right)+k_F\left(A+(1-A)e^{-\alpha t}\right)\ A\alpha=F \tag{14}$$

Combining Equations (13) and (14) gives

$$(1-A)\frac{k_F}{A}=k_F\left(N-1\right)\ A=\frac{1}{N} \tag{15}$$

and so the solution to the system of Equations (4)–(6) is given by

$$\rho_1=\frac{1}{N}\left(1+\left(N-1\right)e^{-Nk_Ft}\right)e^{-\frac{t}{\tau}} \tag{16}$$

$$\rho_i=\frac{1}{N}\left(1-e^{-Nk_Ft}\right)e^{-\frac{t}{\tau}} \tag{17}$$
